# Protection of Water Resources from Agriculture Pollution: An Integrated Methodological Approach for the Nitrates Directive 91–676-EEC Implementation

**DOI:** 10.3390/ijerph182413323

**Published:** 2021-12-17

**Authors:** Carmine Massarelli, Daniela Losacco, Marina Tumolo, Claudia Campanale, Vito Felice Uricchio

**Affiliations:** 1 Water Research Institute, Italian National Research Council (CNR-IRSA), Via F. De Blasio 5, Zona Industriale, 70132 Bari, Italy; daniela.losacco@ba.irsa.cnr.it (D.L.); marina.tumolo@ba.irsa.cnr.it (M.T.); Claudia.campanale@ba.irsa.cnr.it (C.C.); vito.uricchio@ba.irsa.cnr.it (V.F.U.); 2 Department of Biology, University of Bari, 70126 Bari, Italy

**Keywords:** Nitrates Directive, water pollution, GIS, microbial source tracking, Puglia Region

## Abstract

Nitrogen is a vital nutrient helpful to plants and crop growth. However, among the leading causes of water resources pollution is the excess nitrogen from agricultural sources. In European Union countries, the Nitrates Directive has been approved to reduce this problem monitoring of water bodies with regard to nitrate concentrations, designation of Nitrate Vulnerable Zones (NVZs), and establishing codes of good agricultural practices and measures to prevent and reduce water pollution from nitrates. In light of this, we propose an integrated methodological approach to better manage a environmental issue as the perimeter of NVZs with the prospective that our approach could be used in the future by other member states representing a Best Practice in that direction. The methodology is based on data integration applied in a GIS environment. Different available data representing the knowledge of the territory were harmonised, systematised and georeferenced, in order to increase the environmental framework, preserve the contamination of the water resource and give indications on the measures to be implemented to apply in the best way possible the Nitrates Directive. Finally, it was also possible to overcome the infringement procedure in progress for Italy and the Puglia region and proceed to new designation of NVZs.

## 1. Introduction

Water resource quality is vital for the health of humans and natural ecosystems alike; thus, its sustainable management with “ad hoc strategies” is a focal topic for policymakers in both developed and developing countries.

Pressures on water bodies originate from both natural processes (e.g., precipitation inputs, erosion, and climate changes) and anthropogenic activities (urban, industrial, and agricultural) [1]. With its excess nutrients discharge, agriculture is often the predominant worldwide source of water pollution [2].

According to the Food and Agriculture Organization of the United Nations (FAO) report [2], nitrate of agricultural origin is the most common chemical pollutant in the world’s groundwater aquifers; its contamination influences the quality of drinking water resources, with adverse effects on ecological and human health [3].

The nitrate leaching potential from soils is conditioned by several agricultural and environmental factors such as management practices (nitrogen fertiliser application doses and irrigation types), soil properties (soils texture and drainage), regional climatic conditions waste industrial, untreated waste discharge, and sewage spilling [4,5]

In fertilized soils, ammonium is oxidized into nitrites and nitrates, which are easily dispersed from soils into groundwater and surface water bodies. Consequently, the excessive use of fertilizers determines an increase in these nitrogenous compounds, causing environmental and health problems. Moreover, studies have shown that N leaching and nitrous oxide emissions increase when N inputs largely exceed N crop removal [6]. Denitrification is an important process in that it deletes fixed nitrogen—such as nitrate—from the ecosystem and returns it to the atmosphere in a biologically inert form (N_2_). In fact, nitrogen losses for denitrification in agriculture can represent up to 30% of the fertilizer applied [7]. The nitrogen balance (N) provides an indication of the risk of N losses in the environment. However, knowledge of the influence of soil and management factors in N equilibrium is the subject of continuous research.

However, excess nitrates in groundwater might be related to other inputs such as waste industrial, untreated waste discharge, and sewage spilling.

The EU Water Framework Directive (WFD) [8] has set thresholds for achieving good chemical status in groundwater and good environmental status in surface water. Moreover, the identification of pollution sources and a detailed understanding of the processes affecting local concentrations of contaminants is essential for the management of water quality conservation [9].

With Council Directive [10], usually referred to as “Nitrates Directive” (ND), the European Union intervened to ensure that the Member States protect the quality of the water.

In compliance with the Directives, it is necessary to control the concentration of nitrates in freshwater (surface and groundwater) and the eutrophic state caused by nitrogen in surface freshwater, transitional waters and coastal marine waters.

The criticality limit identified by the European Union is 50 mg/l of nitrate, taking into account human health and environmental concerns. This limit has been identified from World Health Organisation (WHO) based on the fact that when nitrate levels in drinking-water exceed 50 mg/L, drinking-water will be the major source of total nitrate intake, especially for bottle-fed infants [11].

Waters with NO_3_ concentrations above 50 mg/L are considered polluted. Since 2011, an intermediate class from 40 to 50 mg/L has also been introduced, reflecting a station “at risk of exceeding the level in the short term”.

If this limit is exceeded in freshwaters, a eutrophic state is registered, or the environmental conditions are such as to believe that—in the short term—the waters can be considered polluted if no action is taken. Cultural eutrophication, defined as the acceleration of nutrient inputs to surface waters caused by human activities, change the amount of nitrate available for primary producers, with algal bloom and tainted drinking water supplies as consequences [11].

Following identification, the relevant territory must be designated vulnerable, and, in this context, agronomic behaviours must be applied that are more attentive to the possible releases of nitrogen into the water (Action Programs).

According to the ND, the Regions must define provisions on the zoning of the regional territory and the following factors concerning:

i. the identification, every four years, of the territorial areas particularly susceptible to being polluted, so-called “Nitrates Vulnerable Zones” (NVZs);
ii. the preparation, within one year from the designation of the NVZs, of a set of mandatory and policy measures (Action Programs) that must be adopted by farmers, especially by those who carry out activities concerning livestock production and practices related to nitrogen fertilisation;
iii. the application, by farmers, of a set of critical interventions for the correct management of agricultural activities to protect the soil and water resource, which must be referred to the Code of Good Agricultural Practice defined by the Ministry of Agriculture and Forestry Policies [12];
iv. training and information initiatives aimed at agricultural operators concerning agronomic practices to protect the environment.

In this context, to comply with the objectives of both the WFD and ND, Member States adopted different strategies. A monitoring system for diffuse pollution from agriculture has been implemented in Estonia. The measured data from small representative catchments in different hydro-geographical regions were used as reference values for areas with no direct measurements for modelling diffuse pollution from agricultural dominated catchments [13].

In Croatia, a novel LUMNAqSoP approach for ND monitoring has been adopted to evaluate the most dominant loads from the agroecosystem and environmental vulnerabilities and sensitivities of groundwater resources to nitrate pollution [14].

The Scottish Environment Protection Agency has developed another new methodology utilising nitrate monitoring data together with several additional lines of evidence to assess confidence in the outcome [15].

It is in this framework that the current study is born, based on the routine activity of Review of Vulnerable Areas from Nitrates of agricultural origin and updating of the related Action Program of the Puglia Region (South Italy) according to an agreement of cooperation between the Puglia Region and the National Council of Research-Water Research Institute (CNR-IRSA) of the city of Bari (Italy).

The main objective of the work is to describe and present the methodological approach adopted in the revision of the NVZs in the Puglia Region. Indeed, despite Directives imposing strict rules and controls on the perimeter of NVZs, methodological instructions on how to dispose of them are not specified. It follows that subjective and different criteria are used to the NVZs perimeter on a global level with resulting un-balanced strategies used worldwide. Our study aims to propose an integrated approach helpful to the Member States as a guideline and a model for a balanced perimeter of NVZs. It considers several aspects of the territory and many networks of data.

## 2. Materials and Methods

The methodology developed draws its origin from the request by the Puglia Region Authority compared to the CNR-IRSA to review the perimeter of the NVZs of the previous four-year period (2012–2015), which were identified only based on the measured values of nitrates in the underground wells monitored.

The public administration also requested the systemisation and elaboration of many available data to increase the quality of the result obtained and achieve a better perimeter of NVZs based on an integrated data approach.

**Figure 1:**
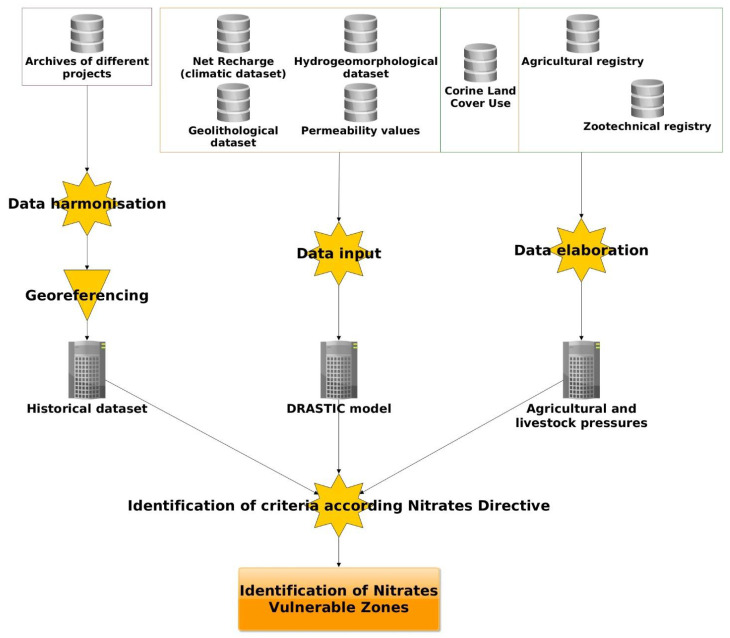
Operational workflow adopted.

In Figure 1, the operational workflow followed for this purpose is shown.

### 2.1. Study Area

The study area is the Puglia Region (Southern Italy). It extends into the north-eastern Mediterranean in the NW–SE direction and constitutes the easternmost part of the Italian peninsula. It has a high territorial discontinuity determined by the considerable development of the coastline and by a highly articulated surface morphology.

Due to its particular geographic position and the accentuated territorial discontinuity, Puglia has highly diversified climatic conditions both within the various regional geographic districts and concerning the Mediterranean macroclimate, from which it is dominated. The Adriatic side is markedly affected by the continental climate determined by the mountainous complexes of the north-eastern sector, and by the extensive plains of Eastern Europe progressively attenuated towards the south by the influence of the eastern Mediterranean. The mountain climate influences the north-west region. The result is a mosaic of remote climates, both mesoclimatic and local [16].

It is on the whole characterised by quite hot and slightly rainy summers and not excessively cold and moderately rainy winters, with low rainfall concentrated during the autumn season. Summer days average temperatures between 25 °C and 30 °C with peaks of over 40 °C on the hottest days [17].

**Figure 2:**
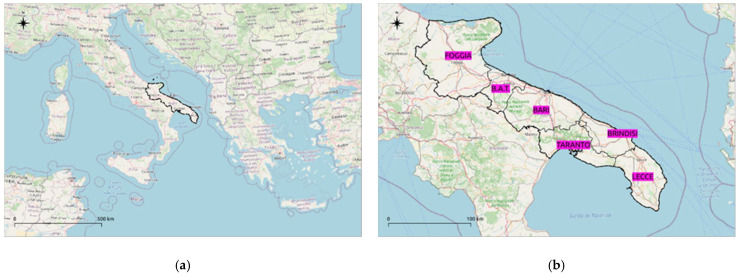
Study area: (**a**) Puglia region (black line); (**b**) the six provinces of the Puglia region: Foggia, B.A.T., Bari, Taranto, Brindisi, and Lecce (violet labels). Base map from © OpenStreetMap contributors.

There is a relevant hydrographical network only in the northern part of the region (Figure 2).

The scarce development of river environments is contrasted by a complex drainage network intended as a particular morphological structure of the territory capable of conditioning the collection and conveyance of surface waters of meteoric origin.

In fact, this drainage network of a fluvial-karst nature is essentially constituted by morphological incisions variously embedded in the substrate, sometimes discontinuous and often without a clear hierarchy. These incisions are generally devoid of any water runoff and affected by substantial and often “ violent ” floods only when certain thresholds of intensity and duration of rainfall are exceeded [18].

Puglia is one of the most important Italian regions for agricultural production, such as olives and vines (not requiring much water and humidity) [19]. It is usually at the top of the national production of oil, wine, and table grapes [20]. In addition, the production of oats and vegetables (artichokes, tomatoes, cabbage, and peppers) is very high [21]. Sheep breeding is very limited, and it seeks to increase the rearing of cattle, still scarce [22]. The fishing is carried out along the coast and, with the big boats, it is also undertaken on the high seas [19,23].

### 2.2. Dataset Reconstruction

The first step of the methodology workflow consisted of the integration of data acquired in several previous projects and monitoring campaigns carried out in the past [24,25]. The total dataset comprises 231 underground water bodies monitoring stations (79 of phreatic type and 152 confined) monitored every six months, and 143 surface water bodies (38 rivers, 6 lakes, 15 transitional waters, and 84 coastal marines) investigated every three months. The data were harmonised by transforming the values with the same unit of measurement and linking them to the acquisition date and the coordinates of the position. By doing so, the data were georeferenced and reported in a Geographic Information System (GIS) system, making it possible to derive the temporal variations with time-series analyses.

A replacement of wells which had become unusable due to various causes that occurred over time was also taken into account. Therefore, based on technical, regulatory updates linked to the correct functionality of the pumping and water extraction systems, data have been merged. About 15 monitoring stations have been merged based on their proximity (< 5 km) and on the knowledge of hydrogeological and hydrogeochemical nature of regional groundwater resources. To this end, a database based on the relational model (RDBMS) written in Structured Query Language [26] was created, which represented the starting point for any other further processing.

### 2.3. Study of the Historical Series Relating to the Concentration of Nitrates

Once the data were made searchable through query languages, the exceedances identified by the ND were calculated [27]. For both surface and groundwater bodies, the number of exceedances for attention threshold of 25 mg/L, alarm threshold of 40 mg/L, and contamination threshold of 50 mg/L were thus identified. Concerning the stations relating to groundwater bodies, a trend was then calculated in cases where the reconstructed series of data could count at least eight values.

The trend value was calculated with the Mann–Kendall test [28,29,30] as recommended by [31,32]. The purpose of the trend calculation is to assess whether there is a statistically significant monotonic upward or downward trend of the variable of interest. A monotonous upward or downward trend means that the variable increases or decreases steadily over time, but the trend may or may not be linear. The Mann–Kendall test can be used in place of a parametric linear regression analysis.

Many authors [33] indicate that this test is the most suitable for exploratory analysis and is used appropriately to identify stations where changes are significant or extensive and to quantify them.

Based on these premises, the historical series relating to nitrate concentrations from 2008 to 2011 and from 2015 to 2018 were examined based on data availability. The concentration exceedances concerning the previously reported thresholds were measured starting from the dataset to understand the level of potential groundwater pollution. In addition, the possible presence and significance of the trend with the test were assessed for each station. The results were reported with the following trend values: “increase”, “decrease”, and “no trend”, with different alpha significance values 0.01 and 0.05; purely as an indication, alpha 0.1 was also calculated only for the understanding of phenomena and trends on a larger scale, highlighting that it is not statistically significant.

**Figure 3:**
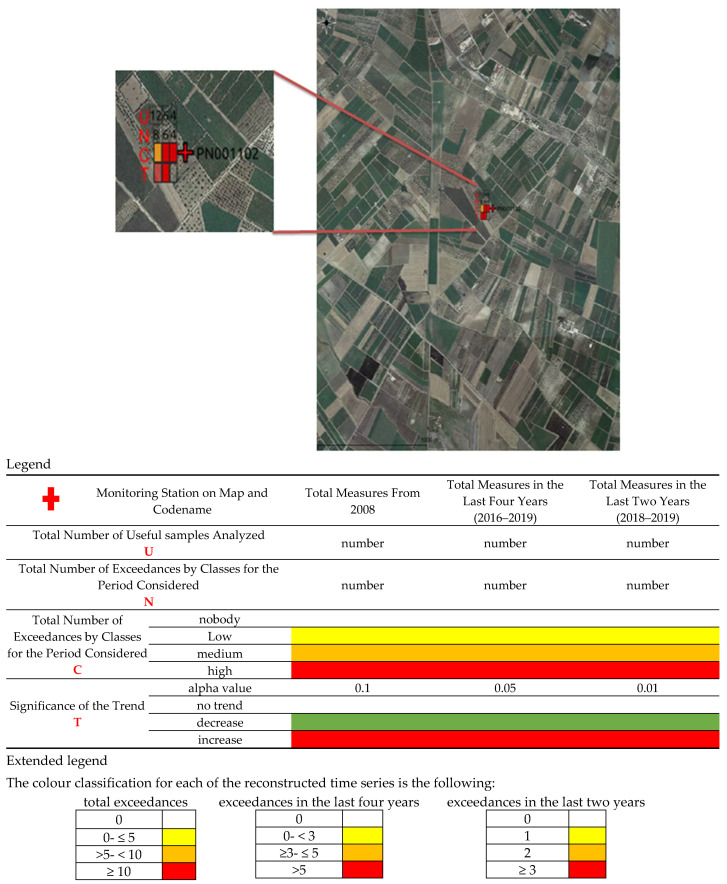
Georeferencing of data and production of synthetic thematic maps for immediate understanding of the status of a monitoring station. In this picture, a groundwater monitoring well is shown (PN001102); it results in a total of 12 (from 2008 to date), 6 (for annuality 2016–2019), and 4 (for annuality 2018–2019) samples analysed, with 8, 6, and 4 exceedances referred to the same periods. The total number of exceedances have been classified and coloured with the value reported in the extended legend. In detail, the exceedances are medium (n. 8 and color orange) for the period from 2008 to date, high (n. 6 and color red) for the period 2016–2019, and high (n. 4 and color red) for the period 2018–2019.

All information was mapped and reported in summary thematic maps, classifying the number of exceedances and the significance of the trends, if any, with different colours. Based on these further elaborations, synthetic thematic maps have been created for water bodies. Below is the georeferencing and classification system of the data adopted and designed to allow rapid identification of the state of the water body in the monitoring station, which can be evaluated through the relative legend (Figure 3). The created geodatabase allowed thus the production of synthetic thematic maps for each single monitoring station.

### 2.4. Estimation of Intrinsic Vulnerability through Modelling Applications Aimed at the Definition of the New Perimeter of the Nitrates Vulnerable Zones

The intrinsic vulnerability of aquifers is defined as the specific susceptibility, in the different geometric and hydrodynamic situations, to ingest and diffuse a fluid pollutant such as to impact on the quality of groundwater, in space and time, even mitigating its effects [34].

The data coming from intrinsic vulnerability play a significant role in the perimeter of the NVZs. They allow identifying areas vulnerable to contamination where the reading of the spatialised data, coming from the monitoring, may not provide all the necessary information.

Therefore, the vulnerability estimated for the aquifers of the Puglia Region was made using a standardised system for evaluating groundwater pollution potential using local hydrogeological settings, the DRASTIC method [35]. The modelling application was implemented based on the reworking of the geo-lithological map drawn up by the Basin Authority of the Puglia Region [36] and the acquisition of meteorological data for the period 1950–2013 provided by the Regional Civil Protection Section [37], as well as the period 2008–2018 by the Agrometeorological Service or Puglia Region [38].

The DRASTIC method considers all the aspects for the estimation of vulnerability whose guidelines have been drawn up by the National Group for Defense from Hydrogeological Disasters [39]. It is applied to evaluate large areas of territory at the same time.

DRASTIC is the acronym of the following seven parameters that are indexed for the calculation of the vulnerability:

- depth to water (D), which is the depth of the aquifer (unconfined aquifer is assumed);
- net recharge (R), which is the net recharge of the aquifer (in the case study, we proceeded by processing the data through the application based on the Thornthwhite method [40,41]);
- average aquifer (A), or lithology of the aquifer;
- soil media (S) to indicate the type of soil;
- topography (T), which is the acclivity of the topographic surface [42];
- impact of the vadose zone media (I), understood as the impact of the unsaturated area, data from the hydrogeomorphological [43] and geo-lithological [36] maps of the Puglia Region were processed;
- hydraulic conducibility (C), understood as the hydraulic conductivity of aquifers, the data of the permeability values were digitised and processed [44].

**Figure 4:**
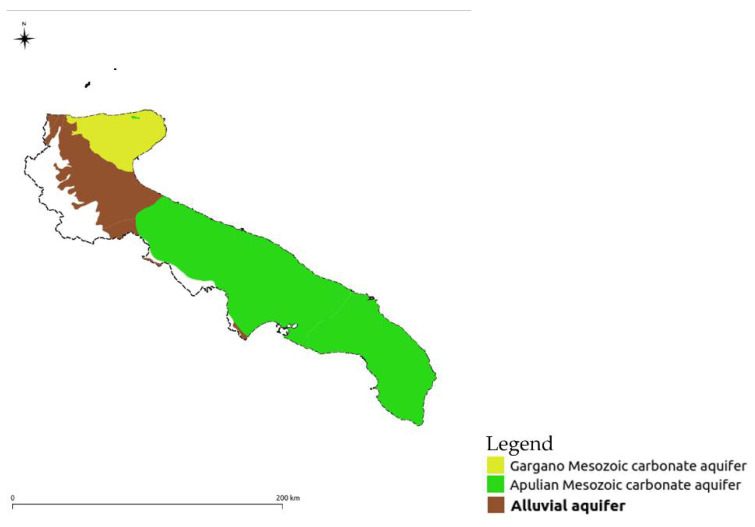
Main subdivision of the aquifers of the Puglia region.

The results from the application of the method were then classified for three different macro-areas (Puglian Mesozoic carbonate aquifer, Puglian Mesozoic carbonate aquifer in the Gargano area, and alluvial aquifer of the Tavoliere) (Figure 4) based on a different attribution of weights related to a detailed knowledge of the territories (Table 1).

The ranges of weights shown in Table 1 refer to aquifers that show different characteristics extensively studied in the context of numerous drilling carried out by the Irrigation Authority and Oil Research Studies. In particular, the ranges for the Mesozoic carbonate aquifer derive from the fact that it is highly permeable due to fracturing and karst and, in coastal areas, highly exposed to the phenomenon of marine intrusion. The Mesozocic carbonate aquifer of the Gargano is more compact, with an underground water circulation present at greater depth in the limestone–dolomite formations and therefore less exposed to contamination phenomena, which is why the ranges differ and the values are low risk. Lastly, the alluvial aquifer of the Tavoliere is characterised by a permeability due to porosity that varies from layer to state, closely linked to the nature of the aquifer mattress (sandy, silty, or clayey) almost comparable to the first aquifer.

The results from applying the method for estimating classified intrinsic vulnerability are reported in the results section.

### 2.5. Pressures on Water Bodies Posed by Agriculture and Animal Farming

Pressures on water bodies related to agriculture and animal farming were considered just in aggregate form at the municipal level, as the available data are not georeferenced, i.e., the detailed company information is not related to geographical coordinates but only to the name of the municipality to which the company belongs.

#### 2.5.1. Estimation of the Pressures on Groundwater due to Agricultural Activity at the Municipal Level

The estimation of agricultural pressure was used to quantify the risk of groundwater pollution caused by nitrates from agricultural sources.

This indicator has involved several components. The components reflect the elements associated with the nitrogen (N) source at the soil surface.

The components used to examine the risks associated with the agricultural activity are:

1. the crop types surveyed in the dataset of the Puglia Region Section Food and Agriculture Sectors in Annex II to the Rural Development Plan [45];
2. the nitrogen uptake, defined as the nitrogen ions absorption by the epidermal and cortical cells of the root [46], and measured in kg/ha of the most widespread Puglian vegetable crops (Table 2);
3. the spread of cultivation measured in ha [47];
4. nitrogen fertilisation practices defined as N application through fertilisers and manures [48].

Subsequently, the demand for nitrogen was calculated for each plant category’s cultivation extension (ha), aggregating data at the municipal level.

Then, the indicator of the nitrogen contamination risk from agricultural sources has been normalised, with a value between 0 and 1, considering the extension of the cultivations towards the extension of the municipality to which it belongs. In such way, we also obtain in a single number the information that contains the distribution of the cultivated areas by municipality.

Based on the knowledge of the area, we considered it appropriate to classify the values in this way and map them on GIS:

- <0.5 = low;
- 0.5–0.8 = medium;
- >0.8 = high.

the classification adopted for the values from 0 to 1.

#### 2.5.2. Estimation of the Animal Farming-Induced Pressure on Groundwater at the Municipal Level

The animal farming-induced pressure on groundwater, attributable to the run-off or leaching of manure, was also estimated. To this aim, the stocking density was considered a risk factor and was calculated as the ratio between the total amount of livestock units (TLU) and the available grazing area (GA) at the municipal level.

The number of bovines, sheep, goats, pigs, breeding sows, hens, and other poultry bred in each municipality was extracted from the Puglian Register of Animal Breading dataset [49], and then multiplied by the coefficient set in EU legislation for animals conversion to LU, according to the annex V referred to the EU L 368, in article 27(13) (Table 3) [50].

TLU values, obtained as mentioned before, might in some cases represent an overestimation, as it was assumed that each animal was over six months, or, in other cases, underestimation due to the lack of livestock detailed information.

Information about the GA extension was derived from the Puglian Land Use database (up to the four levels of classification), updated to 2011 [51].

TLU/GA ratio was calculated, and then resulting values were normalised in a range between 0 to 1. Values above 0.8 were considered indicative of areas potentially at high risk of nitrates contamination from faecal pollution.

The corresponding risk map was generated in GIS environment and used, together with the risk map of potentially nitrogen contamination from agriculture, as a decision-supporting tool to orient further investigations. 

### 2.6. Criterion for the Selection of Water Monitoring Points Functional to the Identification and Correct Perimeter of Nitrates Vulnerable Zones

The water monitoring points were selected in which critical issues were found in the last four years of monitoring (2015–2019) based on the information now available to which the following criteria were applied:

- at least an excess of 50 mg/L in value in the last four years following an extremely precautionary approach;
- average in the last four years between 40–50 mg/L with an increasing trend;
- water monitoring points already in NVZs but for which there have been no samplings in the last four years;
- However, the points with the following criteria were not selected:
- average in the last four years between 40–50 mg/L with a stable to decreasing trend;
- average in the last four years between 25–40 mg/L with only one single measure more than 50 mg/L, stable or decreasing trend, in the previous data there is no other overcoming of the threshold;
- average in the last four years between 25–40 mg/L with only one single measure more than 50 mg/L, a trend not calculable (less than eight measures) with both low values of agricultural and livestock pressures;
- average in the last four years between <25 mg/L with only one single measure more than 50 mg/L, stable or decreasing trend;
- average in the last four years between and maximum measured value <40 mg/L;
- water monitoring points located in urban centres.

Applying these criteria, *n* = 156 water monitoring stations relating to groundwater wells have been selected.

Another aspect should be added. Recently, at the national level, a ministerial working group has drawn up a document containing the guidelines defining the trophy in compliance with 2000/60/CE, 91/676/CEE, and 91/271/CEE. The methodology reports the criteria for evaluating eutrophication that should be applied on an experimental basis in all Italian regions. This methodology considers the chemical-physical properties monitored and also considers the biological ones, in particular of the plant components (macrophytes and diatoms) susceptible to nutrient variations. The trophic class (E1, E2, or E3) is obtained from the intersection between the information on the quality class defined by the LIMeco index [52] with the quality class defined based on the diatoms calculated with the ICMi index [53] and of the calculated macrophytes with the IBMR index [54], according to the following scheme (Table 4):

Therefore, the sites for which at least one of the following conditions are verified are inserted to be protected: average nitrate values > 40 mg/L, maximum values > 50 mg/L, and in which Table 4 indicates an E1 value. In total, there are 25 points.

### 2.7. Performance of Faecal Bacteria Tracking Combined to GIS Database to Discriminate Nitrate Source

Geographic Information Systems (GIS) are recognised as an innovative tool managing geographic information holistically without losing space-time variability. This approach integrates common database operations and statistical analysis, providing a support tool in the management of water quality definition. Using an integrated molecular approach, it is possible to describe the main sources of agricultural contamination where the GIS system highlights areas subject to different origin contamination.

Microbial source tracking (MST) is a molecular method to determine sources of faecal contamination via detecting gastrointestinal bacteria by polymerase chain reaction. MST allows for the identification of probable animal sources contamination [55,56,57,58,59,60]. Species of the order *Bacteroides* have been identified as indicator microorganisms of faecal contamination, as they may be species-specific or group-specific and associated with recent contamination [61]. In detail, the amplification of 16S rRNA genes of host-specific *Bacteroidetes* allows discrimination against human and livestock faecal sources in samples from nitrate polluted environments.

The ammonia oxidants’ physiological characteristics subgroup allowed them to be used as an indicator of the environmental change associated with using nitrogen chemical fertilisers. 16S rRNA gene has been proposed as a molecular marker for agricultural pressure [62]. The identification of nitrate contamination resulting from the excessive application of mineral fertilisers, the group of microorganisms ammonia-oxidizing bacteria (AOB) is a valid biomarker for assessing contamination source [63]. The functional gene coding for the subunit ammonia monooxygenase (*amo*A), found only in AOB, has proved useful as a specific molecular marker in environmental studies [64]. The present work evaluated the performance of eight microbial source tracking (MST) and bacterial markers in 16 groundwater samples. All samples were tested with human [57,65,66], cattle [55], pig [67], equine [67], and amoA [63] markers by PCR and quantitative PCR (qPCR). Internal validity metrics were calculated using faecal samples and fertilised soils. Further details regarding the matherials and methods adopted are shown in Supplementary Materials.

### 2.8. GIS Methodology for the Perimeter of Nitrates Vulnerable Zones from Agricultural Sources for Groundwater and Surface Water Bodies

Once the subset of data functional to the new perimeter of vulnerable areas was identified, the areas that could be represented by the point concentration value detected in the measurement station were identified through topological overlays.

**Figure 5:**
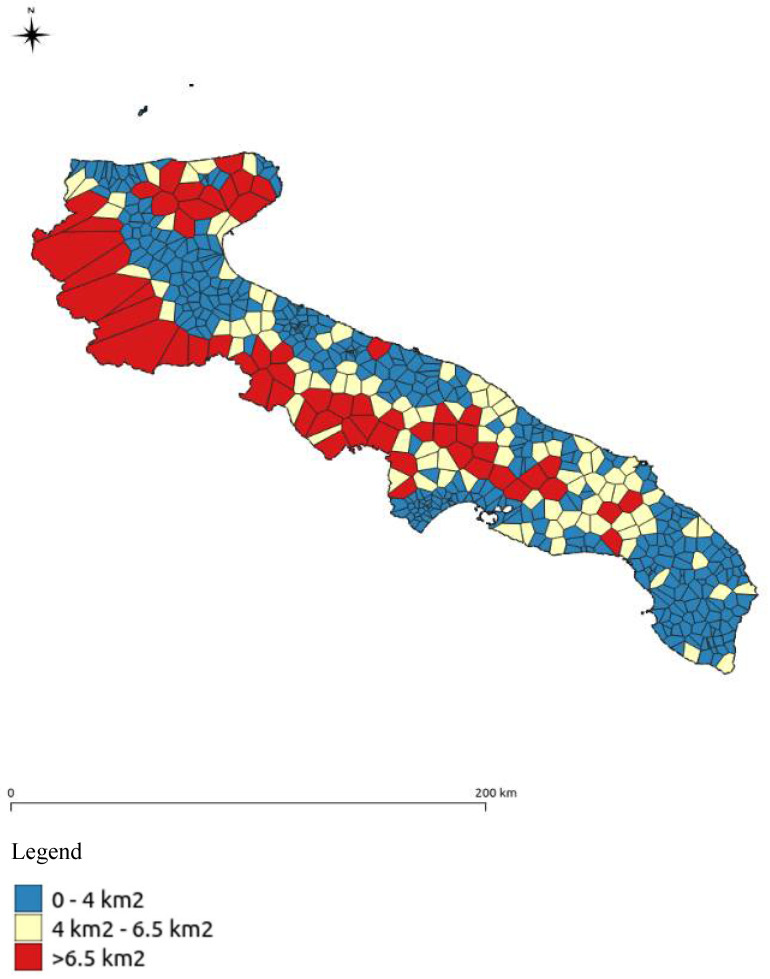
Subdivision of the Puglian territory into polygons of influence and classification based on their extension.

As regards the underground water bodies, the entire regional territory was first divided into polygons of influence [68], referring to the methodology provided by the Higher Institute for Environmental Protection and Research (ISPRA) in the guidelines for the selection of areas to be remediated as part of the characterisation of contaminated sites [69]. Subsequently, the polygons that had an extension < 4 km^2^ (based on knowledge of the territory) were selected as valid (the blue polygons in Figure 5). While for the red and yellow polygons, given that the parameter selection did not suggest an adequate representation of the areas in reference around the point, a 2 km^2^ buffer was created around the monitoring station to identify the area of interest.

**Figure 6:**
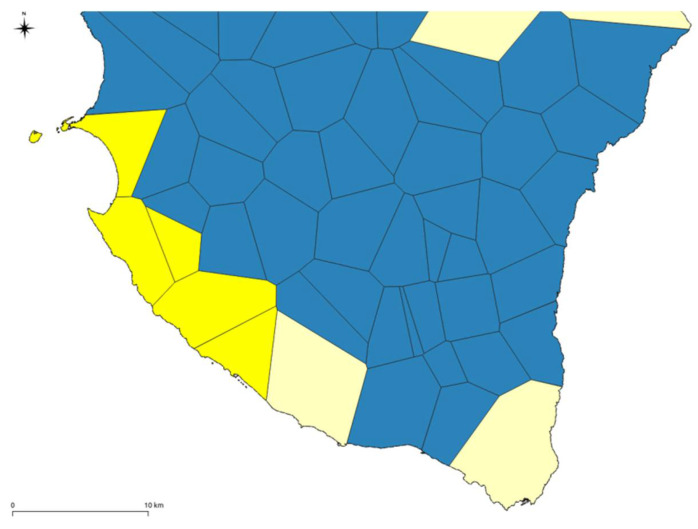
Selection example of polygons where wells resulted polluted from nitrates of agricultural origin.

The areas above the groundwater wells identified with the aforementioned criteria, based on chemical analysis and biomolecular approach, have been figured and selected below. An example of this phase is shown in the following Figure 6.

**Figure 7:**
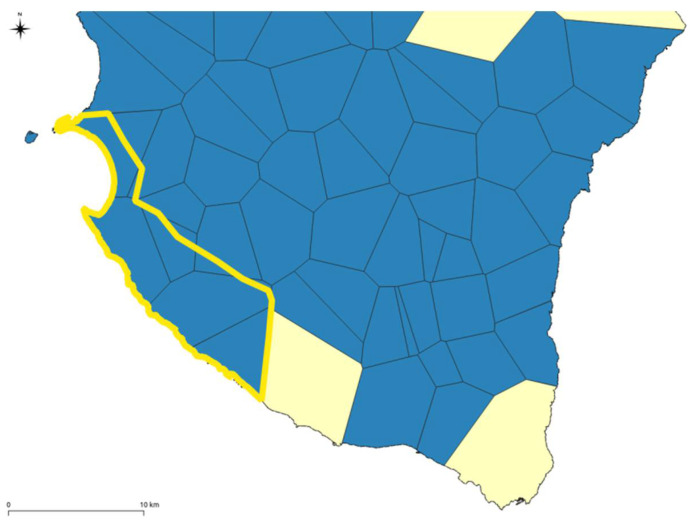
Example of simplification resulted by the selected geometries of Figure 6.

The selected areas (yellow colour in Figure 6) have been merged, and the geometries simplified [70] to identify a certain continuity in the territory by comparing these perimeters with the topography [42] (Figure 7).

**Figure 8:**
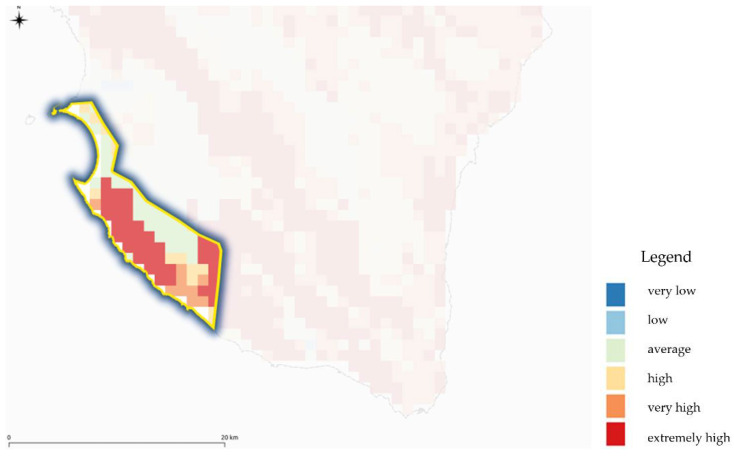
Example of continuity-discontinuity assessment of the vulnerability value in the selected areas obtained by DRASTIC model application.

After this simplification phase, we proceeded with the analysis of other available information: the contiguity of areas was assessed from time to time based on intrinsic vulnerability (the entire map will be shown in the results section) (Figure 8), the prevailing direction of the flow with the isophreatic, the values of the calculated pressures, the proximity of urban centres and sewer pipes, and the comparison with the cave cadastre [71] to assess any contamination due to illegal disposal, as well as the continuity of the environment and ecosystems through assessments with the Natura 2000 sites [72].

**Figure 9:**
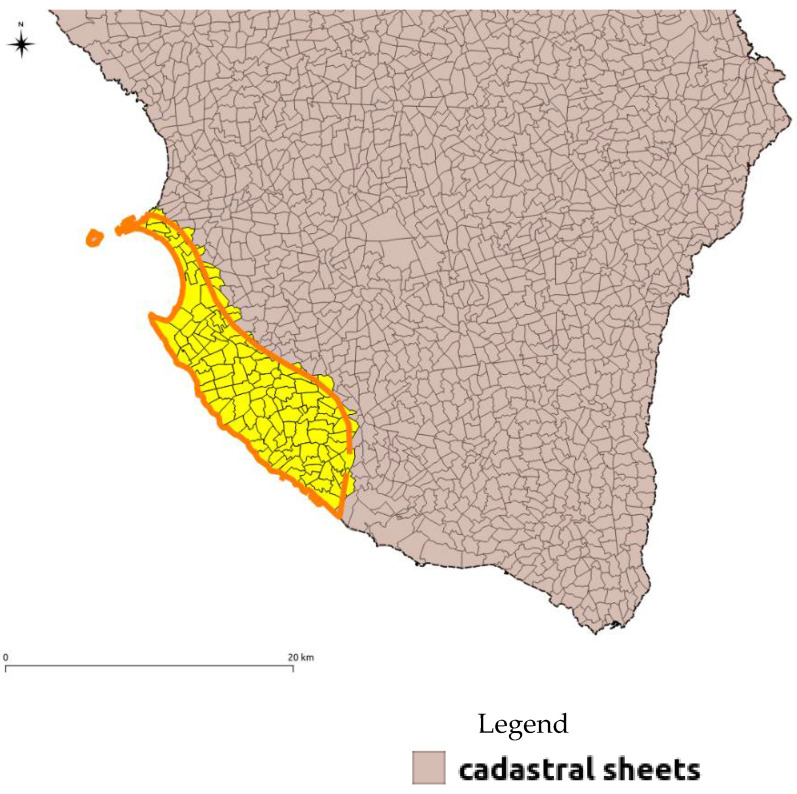
Example of final perimeter of groundwater bodies with identification of cadastral sheets for a simplified administration of the NVZs.

The areas to be perimeter were thus defined, and subsequently, the cadastral sheets, which overlapped by more than 30% on the areas identified, were extrapolated (Figure 9). This choice was made to facilitate the management of these areas from an administrative point of view.

**Figure 10:**
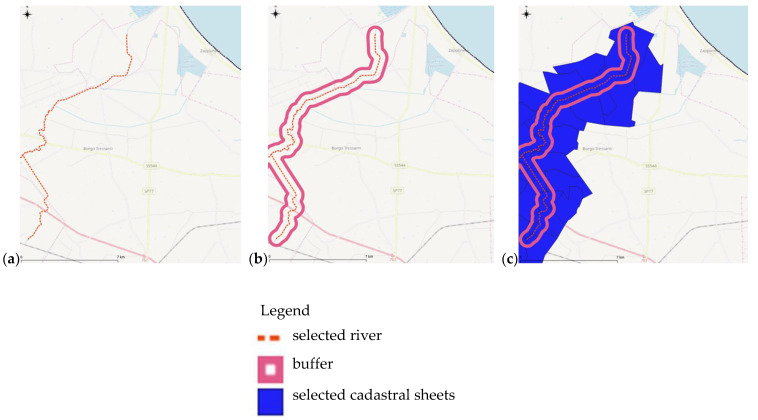
Example of the final perimeter of surface water bodies with identification of cadastral sheets for a simplified administration of the NVZs. (**a**) Identification of the river; (**b**) creating the buffer; and (**c**) topological overlay with cadastral sheets.

Regarding the surface water bodies, a buffer of 1 km was first identified in reference to the river, and then the cadastral sheets, which overlap more than 30% on the areas, were selected to simplify the administrative management of such areas. In the residual areas between the various NVZs, the perimeter activity was guided by analysing the agriculture and animal farming pressures (Figure 10).

Not considering the normal hydrographic limits makes the administrative management of the areas easier; also, in this case, the precautionary criterion and verifying the limits of the perimeter areas with the limits of the watershed is applied. However, this activity is left to the ability of the researcher to know how to discriminate. Applying the perimeter, considering only the watershed lines, would have practically resulted in a perimeter of the entire Puglia region; obviously, this is incompatible with the ordinary management of the agriculture sector. The perimeter does not consider any information obtainable about the impact of seasonality on nitrogen distribution or availability, as, from a management point of view, it would not be possible to differentiate the areas based on seasonal properties.

## 3. Results

**Figure 11:**
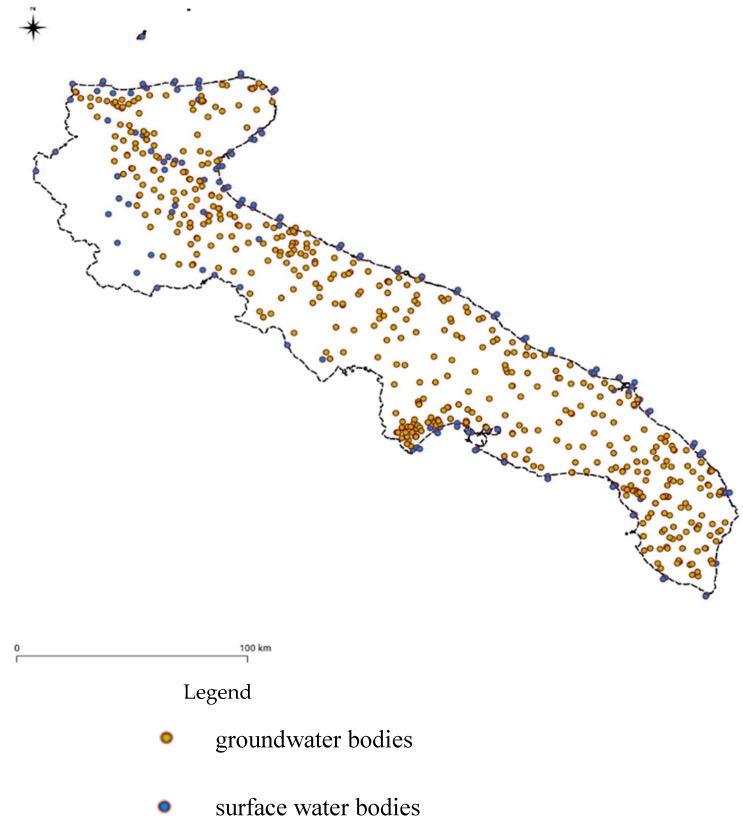
All selected monitoring stations subdivided in groundwater (yellow dots) and surface water bodies (blue dots).

The results concerning the data harmonisation and georeferencing are shown in Figure 11. The dots indicate the monitoring stations where it was possible to recover valuable data to elaborate the historical series activity at the basis of the methodology.

### 3.1. Results from the Historical Series Analysis

The results of the historical series relating to the concentration of nitrates are reported in the following tables. In summary, a breakdown of the data by classes and significant trends is shown for all wells monitored in the last four years (2016–2019) (Table 5 and Table 6):

### 3.2. Results of the Application of the DRASTIC Model

**Figure 12:**
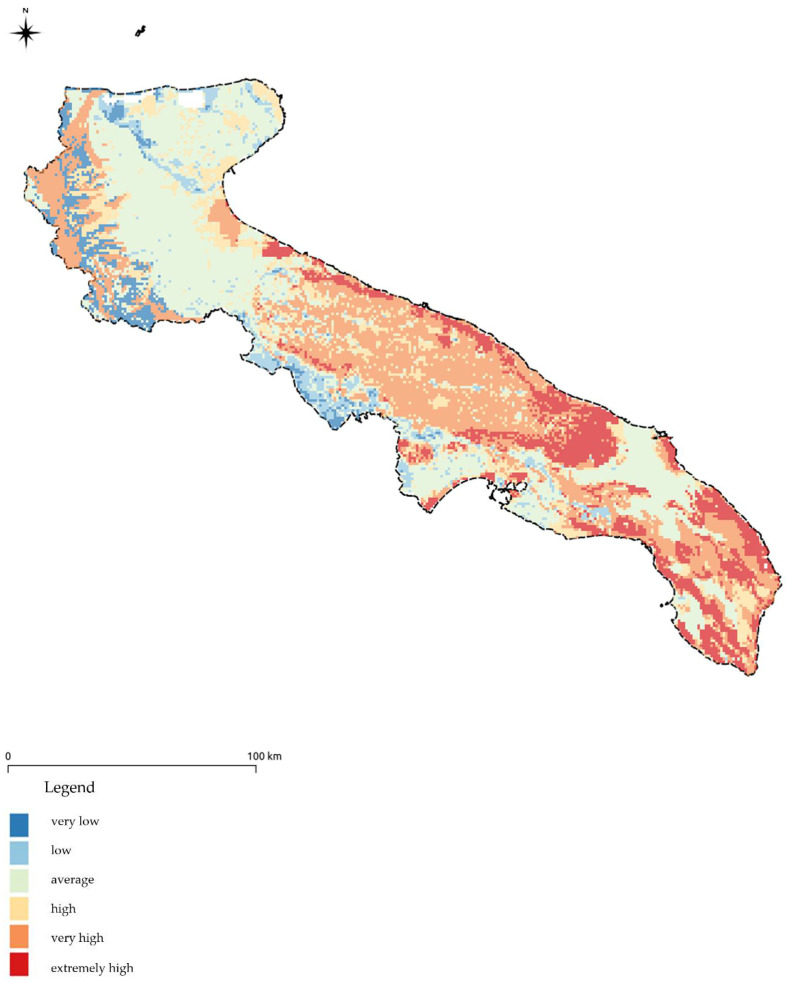
Values of intrinsic vulnerability calculated through the application of the DRASTIC model updated to 2019.

Figure 12, shows the results obtained from applying the DRASTIC model reported in the summary map (GRID files with 1km mesh) classified for a quick understanding.

### 3.3. Map of the Results Deriving from the Calculation of the Pressures on Water Bodies Posed by Agriculture and Animal Farming

**Figure 13:**
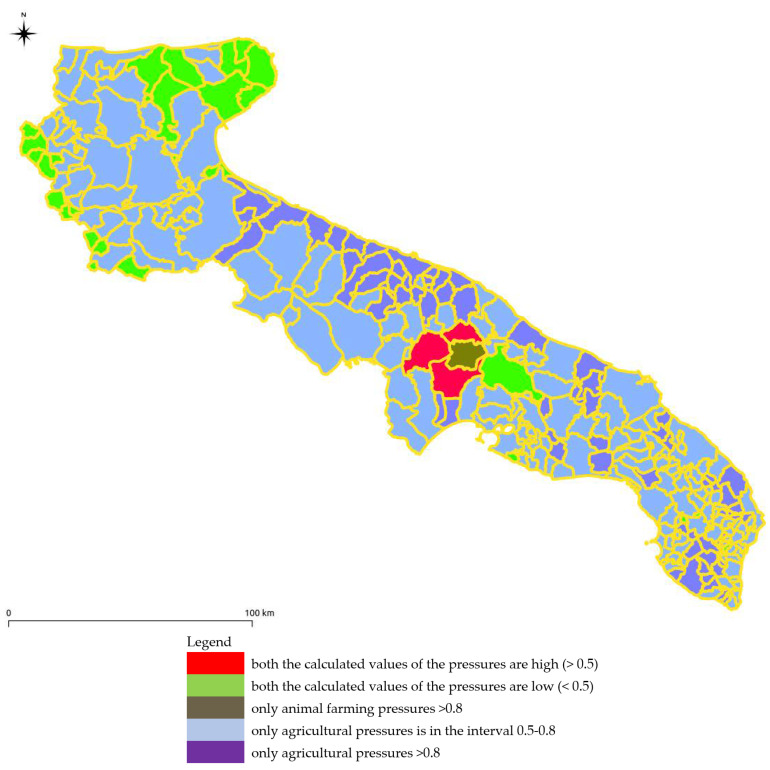
Results deriving from the calculation of the pressures on water bodies posed by agriculture and animal farming.

All information relating to the estimation of the pressure was imported on a GIS system to create a map carried out at the municipal level (Figure 13). Through the classification on the GIS system, it is possible to identify the areas in which both the calculated values of the pressures are high (>0.5) or low (<0.5). The areas with high-pressure values only for the livestock sector (>0.8) are limited while considering only the agricultural sector both the areas with high-pressure values (>0.8) and those with average values (0.5–0.8) are prevalent.

### 3.4. Performances of Microbial Source Tracking Marker Assays

The MST methodology was applied to *n* = 16 groundwater samples collected in the study area from wells considered as “priority”, due to the concomitance of both agricultural and animal breeding-induced pressures, evidenced by the calculation described in Section 2.

PCR-tested biomarkers could not discriminate the principal source of nitrate pollution due to a small number of microbial cells characterising the deepest water samples. In addition, the PCR tests conducted with the primers set specifically for human *Enterococcus faecalis* (Table S1) on positive faecal control were not able to discriminate the zootechnical source from the human one.

On the contrary, animal and human faecal markers were widely detected (using the primers sets qBac560F/qBac725R and HF185F/qHF183rnew, respectively) on eleven groundwater samples, through Real-Time PCR (qPCR). This more sensitive technique, allows the detection of specific signals starting from a few copies of the molecular target. A qPCR experiment carried out using amoA-1F/amoA-1R primers evidenced early amplification signals (Ct) in water samples where a mineral fertilisation-induced pressure was prevalent. The estimation of the relative abundance of the molecular target in samples vs. positive controls at known concentration was conducted through the Ct comparison. This approach allowed us to understand if agriculture-induced pressure or human/animal faecal pollution was the main contribution to nitrates concentration in groundwater samples.

### 3.5. Final Boundary and Estimate of the Variations over the Years of the NVZs Areas

**Figure 14:**
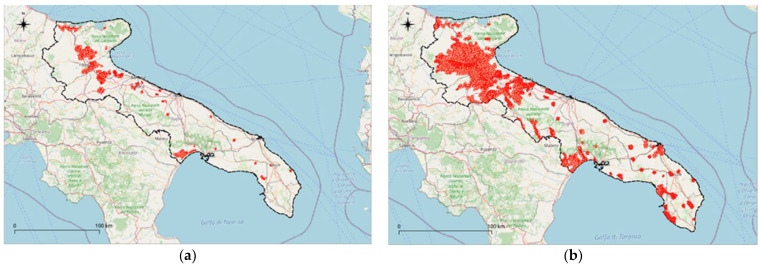
Perimeter of NVZs in Puglia region. (**a**) perimeter of the previous period (2012–2015); (**b**) updated perimeter (27 October 2021). Base map provided by “© OpenStreetMap contributors”.

In Figure 14, it is possible to observe the overall result of the redefinition of the NVZs on the Puglian Region territory following the methodology approach adopted.

It should be noted that, in some areas for which it was not sure that the contamination recorded was attributable exclusively to the agricultural/livestock world, a precautionary perimeter has been chosen.

The future integration of further biomolecular investigations and the refinement of the method will reassess these situations.

Below is a summary indicating the territorial extension of the perimeter areas on a provincial basis and a comparison with the previous designation (Table 7).

## 4. Discussion

The obtained results show an evident growing nitrate pollution phenomenon in the water bodies of the Puglian territories, encouraging urgent actions to protect water resources mainly from nitrates contamination of agricultural origin.

Given that the phenomenon does not seem under control, at the moment, the priority is to deepen the quantitative contribution of the concentrations of pollutants from different sources.

It has to be considered that the estimation of the animal farming-induced pressure on groundwater at the municipal level could not provide a faithful depiction of the reality due to the lack of available database. Particularly, TLU values might represent, in some cases, an overestimation, as it was assumed that each animal was over six months, or, in other cases, an underestimation due to the lack of detailed livestock information (i.e., livestock number and age). In addition, creating a georeferenced database of farms, including attributes related to the property extension, would allow more accurate estimation of the pressure on groundwater given by the stocking density.

The molecular approach developed, taking advantage of the MST strategy, helped identify which suspected nitrate sources significantly contribute to the pollution observed in groundwater monitoring stations in Puglia region.

As nitrate originates from various sources, any monitoring program needs to track and apportion the NO_3_− origin in the contaminated aquatic systems [73]. This information will provide evidence and valuable information regarding the control of NO_3_− contamination, improving water quality, sustainable water resources management, and targeted control measures of contaminated areas.

The last decade has seen the development of numerous methods based on molecular MST of faecal indicator bacteria that have also been developed and tested in water quality management studies, becoming increasingly common [74,75,76].

The main reactions that control the dynamics of nitrogen in soil and groundwater are mineralisation, volatilisation, nitrification, and denitrification, for the most part, mediated by microorganisms that play a key role in the fate of nitrogen in terrestrial ecosystems. [77,78]. Therefore, monitoring the microbial communities involved in the nitrogen cycle represents a valuable tool for determining potential sources of nitrates affecting surface and groundwater.

The results of molecular methods indicate that biomarkers can be considered reliable in distinguishing the nitrate pollution origin. Nevertheless, our study shows that the experiments conducted with the human *Enterococcus faecalis* biomarker do not discriminate the zootechnical source from the human one. In addition, in some cases, PCR-tested biomarkers cannot determine the source of contamination in environmental matrices due to the detection limit.

Another critical point is that nitrogen fertilisation determines an increase in ammonia-oxidizing bacteria (AOB) abundance in environmental matrices. However, the response of AOB abundance to fertilisation depends on some factors, such as the land-use types [79].

MST technology demonstrated to be a useful tool for local authorities to detect the nitrate contamination source and review uncertainties during the NVZs definition and the action program development required by the European Nitrates Directive.

In addition, it seems necessary to have more detailed data, at least at the parcel level (which is a small cadastral unit), to make the mapping more precise.

The proposed approach integrates a chemical monitoring of nitrates pollution with a molecular strategy (MST) intended to discriminate between human and animal sources of faecal contamination in the aquatic environment. The integration of these methodologies has provided useful answers both in the application phase of models for the perimeter of the NVZs and in the subsequent monitoring plans of the Puglia region. The aim is to better orientate the implementation of the Plan measures according to the different pressures, understanding the dynamics that govern the release of nitrates into the environment.

Another aspect that we consider almost necessary is the possibility of implementing a registry of controls and an online database of the Agronomic Plans for the use of sewage. Thus, it would be possible to achieve a high level of knowledge for the whole regional territory, making the identification of any unsustainable practice causing nitrate pollution relatively easy. In addition, measures to back the realisation of consortium and purification plants treating zoo-technical waste and strategies to support the synergy in regional planning should undoubtedly be indicated.

The GIS-based approach adopted in the present work allowed the integration of different data sources to optimise the available information; for example, the inputs provided by the biomolecular activities were very useful to discriminate the perimeter of the NVZs. The benefits increase with more detailed data, such as the number of head of cattle for each type of farm with information on their positions and extensions of grazed fields, allowing a more detailed perimeter in GIS environment.

The support provided from GIS environment in this application is vital to allow the management of environmental big-data and immediately understand the phenomena in progress to achieve better management by territories administrators. Considering the “easy to use” strategy adopted to develop the present methodological approach, a significant value of our workflow is undoubtedly its possible adoption by other Member Stated as a model to follow to obtain comparable and standardised results at a European level.

For example, in the regions near Puglia, perimeters are based on the entire hydrographic basins, creating an excessive discontinuity, a problematic management of border areas, and a complex understanding of the environmental phenomena in progress.

Furthermore, by exploiting the most advanced communication and processing standards, it is possible to share information among different systems to facilitate decision-making by aggregating data from heterogeneous sources.

Moreover, if we add the potential of the interoperability standards that can be implemented for which the Guidelines [80] have recently been adopted by the Italian Agency for Digital Innovation, we can positively consider setting up this methodology that can be easily adopted by several Member States that face this phenomenon, increasing efficiency and use of GIS.

Finally, by implementing a global policy in the use of GIS systems, it will be possible to raise awareness of environmental issues by involving all economic bodies to achieve sustainable development, which, to date, still represents a fundamental element of the principles established in Rio during the Earth Summit [80]. Awareness of the problems and options available is of decisive importance: information, education, and training on environmental management are fundamental elements for sustainable development. They can be achieved by disseminating research results, integrating environmental programs into vocational training, schools, higher education, and adults, and developing networks within economic sectors. All these aspects inevitably converge towards the promotion and participation of the public in decisions through the mechanism of public consultation so that a greater sense of belonging and sharing of responsibilities can emerge.

## 5. Conclusions

During the last decade, the interest aroused by the science of Geographic Information and the success obtained by GIS technologies has strongly encouraged the growth of new fields of applications followed by the development of advanced technologies. These, both hardware and software, are suitable for satisfying the most varied requests from the world of scientific research and from that of territorial planning and environmental protection. All this has favoured the birth of an interdisciplinary dialogue between the technical managers of digital Geographic Information and various users, public and private, who are responsible for managing the territory with all the environmental, social, and economic implications that this entails. The GIS, due to its versatility in data of different nature processing and managing, is an indispensable tool to study the environmental and socio-economic aspects of the territory and the planning and monitoring of interventions.

By applying the methodology described in this work based on a multidisciplinary approach, it was possible, through more detailed knowledge of the territory, to identify the territorial criticalities and consequently provide urgent containment measures as the mapped areas are increasing. Consequently, it emerged that there is a necessity to increase controls in those areas where the incidences are increasing the most. Moreover, it is fundamental to digitise all the agricultural practices applied as much as possible, in order to be able to identify any Worst Practices with surgical precision to preserve high-quality levels of our water resources.

Furthermore, it was also possible to overcome the infringement procedure No. 2018/2249 is in progress for Italy and the Puglia region, setting up all the necessary alternative stations to ensure that the old suppressed points continue to be monitored.

It was thus possible to proceed to new designations to ensure that the corresponding NVZs were designated for all the monitoring stations polluted.

In the event of exclusion of a station, the significant contribution of agriculture was assessed to conserve and improve the quality of soils and water resources, increase the state of wild fauna and flora, habitats, and landscapes.

## Figures and Tables

**Table 1:** Classification adopted by the application of the DRASTIC model for the three macro-areas aquifers of Puglia Region (shown in the table by adimensional numbers).

Classification	Puglian Mesozoic Carbonate Aquifer	Puglian Mesozoic Carbonate Aquifer in the Gargano Area	Alluvial Aquifer of the Tavoliere
Very low	<75	<90	<70
Low	75–90	90–110	70–80
Medium	90–110	110–130	80–100
High	110–130	>130	100–140
Extremely high	>130		>140

**Table 2:** Nitrogen uptake in the most widespread vegetable crops in the Puglia region [45].

Land Cover	Vegetable Crops	Nitrogen Uptake (Kg N/ha)
Permanent crops—Olive groves	Olive trees	140
Permanent crops—Vineyard	Wine grapes	120
Permanent crops—Vineyard	Table grapes	150
Permanent crops—Citrus groves	Orange, clementine, mandarins trees	150
Permanent Crops—Drupaceous	Apricot, peach, plum trees	150
Permanent crops—Drupaceous	Cherry, almond trees	100
Arable land—Cereals	Wheat, barley, oats, rye, soft wheat	100
Arable land—Fodder	Grassland (meadows)	60
Arable land—Fodder	Grassland (pasturage)	40
Arable land	Potato	120
Arable land	Tomato	120
Arable land	Artichoke	120
Arable land	Beetroot	100
Arable land	Umbelliferae family	140
Arable land	Brassicaceae family	120
Arable land	Salad	140
Arable land	Cucurbitaceae family	180
Arable land	Other horticultural species *	145

* Plant species present in the Puglia region not associated with any specific category (legumes, onions, or vegetables not specified). The required nitrogen value is given by the average nitrogen demand of Umbelliferae, Brassicaceae, salad, and Cucurbitaceae.

**Table 3:** Table of conversion of animals to livestock units from EU L 368, article 27(13) adopted in the present work to estimate the animal farming-induced pressure on groundwater.

Category	Livestock Unit (LU)
Bulls, cows, and other bovine animals over two years, equine animals over six months	1.0
Bovine animals from six months to two years	0.6
Bovine animals below six months	0.4
Sheep	0.15
Goats	0.15
Breeding sows > 50 Kg	0.5
Other pigs	0.3
Laying hens	0.014
Other poultry	0.003

**Table 4:** Scheme for the identification of the trophy state.

		Biological Index				
		Bad	Rare	Enough	Good	High
Chemical/physical index	bad	E1	E1	E1	E3	E3
	rare	E1	E1	E1	E3	E3
	enough	E1	E1	E2	E3	E3
	good	E3	E3	E3	N	N
	high	E3	E3	E3	N	N

**Table 5:** Average nitrate concentration values per number of stations.

Nitrate Concentration Values in mg/L (Average of the Last Four Years 2016–2019)	Number of Monitoring Stations
0 < NO_3_ < 25	125
25 ≤ NO_3_ < 40	59
40 ≤ NO_3_ < 50	28
NO_3_ ≥ 50	71

**Table 6:** Test result for the calculation of significant trends by station.

Significative Trend (Alpha 0.05 over the Entire Time Series)	Number of Monitoring Points
decrease	10
stable	148
increase	25
not calculable (<8 values)	100

**Table 7:** Summary tables of the new perimeter of Nitrates Vulnerable Zones year 2019.

Province	Previous NVZs Extension (ha) (2012–2015)	Updated NVZs Extension (ha) (2021)	Impact on the Total Area (%)	Variation (%)
BARI	3535	25,653	6.7	+5.8
BAT	8290	54,851	35.9	+30.5
BRINDISI	738	18,565	10.1	+9.7
FOGGIA	75,707	280,338	40.3	+29.4
LECCE	3578	35,374	12.8	+11.5
TARANTO	12,207	38,124	15.7	+10.7
PUGLIA REGION	104,055	452,905	20.2	+16.3

## Data Availability

All results can be downloaded from the Puglia Region portal: http://www.sit.Puglia.it/portal/portale_cis/Zone%20Vulnerabili%20da%20Nitrati/Perimetrazione%20e%20Designazione (accessed on 16 December 2021).

## Supplementary Materials

The following are available online at https://www.mdpi.com/article/10.3390/ijerph182413323/s1, Table S1: Primers set used to identify the source of nitrate contamination.
