# Supplementary material for: Protection of Water Resources from Agriculture Pollution: An Integrated Methodological Approach for the Nitrates Directive 91–676-EEC Implementation
Source: Int J Environ Res Public Health. 2021 Dec 17;18(24):13323. doi: 10.3390/ijerph182413323 (PMC8704299; doi:10.3390/ijerph182413323)
Supplement: Supplementary file 1 [file ijerph-18-13323-s001.zip › ijerph-1513273-supplementary.pdf]

Supplementary Material

Protection of water resources from agriculture pollution: an integrated methodological approach for the Nitrates Directive 91-676-EEC implementation

Carmine Massarelli [1,*], Daniela Losacco [1,2], Marina Tumolo [1,2], Claudia Campanale [1] and Vito Felice Uricchio[1]

[1] Water Research Institute—Italian National Research Council (CNR-IRSA), Via F. De Blasio 5 - Zona Industriale, 70132 Bari, Italy; daniela.losacco@ba.irsa.cnr.it (D.L); marina.tumolo@ba.irsa.cnr.it (M.T.); Claudia.campanale@ba.irsa.cnr.it (C.C.); vito.uricchio@ba.irsa.cnr.it (V.F.U.)

[2] Department of Biology, University of Bari, 70126 Bari, BA, Italy

* Correspondence: carmine.massarelli@ba.irsa.cnr.it (C.M.)

Materials & Methods

Performance of faecal bacteria tracking combined to GIS database to discriminate nitrate source

Sample collection

The study was carried out during the Apulia Region Nitrate Vulnerable Zones definition. A total of 16 groundwater samples were collected at a depth between 8-365 cm and transported to the laboratory at four °C. Samples were analysed within 24–48 h after collection. Molecular method validation was performed on groundwater bodies that showed high anthropogenic pressures in a GIS environment.

Water samples and DNA extraction

5L of water were filtered on a vacuum manifold through a 47 mm polycarbonate filter with a 0.22 µm pore size. Three filter replicates were collected for each sample. DNA was extracted using the Qiagen DNeasy PowerSoil following the manufacturer's instructions.

Marker identification and 16S rRNA gene amplification

Two different approaches were used to validate the biomarkers' performance: Polimerase Chain Reaction (PCR) and quantitative real-time PCR (qPCR) assays. For both PCR and qPCR experiments, selected primer sets were checked using faecal samples of known origin as positive controls. As shown in Table 4, samples were screened, amplifying a portion of the 16S rRNA gene of eight specific genetic markers via PCR and qPCR.

All PCR reactions were conducted with HotStartTaq Master Mix kit (Qiagen, Germany) with a specific annealing temperature for each biomarker (Table S1). The PCR products were examined by agarose gel electrophoresis. Real-time PCRs were performed using a QuantiNova SYBR Green PCR Kit (Qiagen, Germany) in a Rotor-GeneQ thermocycler (Qiagen, Germany) equipped with the Rotor-Gene Q Series Software. In order to distinguish between main and secondary possible nitrate pollution sources, the samples cycle threshold (Ct), i.e. the calculated cycle number at which the qPCR product crosses a threshold of detection, were compared against Ct values of positive faecal controls at a known concentration. For the

quantification of *amoA* DNA sequence, standard curves were obtained from serial dilutions of a known concentration of plasmid DNA. Standard curves were produced by tracing threshold cycles against 16S rRNA copy numbers.

Table S1. Primers set used to identify the source of nitrate contamination.

Target organism	Primer name	Method	Annealing Temperature (°C)	Reference
Human-*Bacteroides*	HF183F Bac708R	PCR	54	[54]
Cattle-*Bacteroides*	CF128F Bac708R	PCR	53	[52]
Pig-*Bacteroides*	PF163F Bac708R	PCR	53	[64]
Horse-*Bacteroides*	HoF597F Bac708R	PCR	54	[64]
Human-*Enterococcus faecalis*	FL1 FL2	PCR	55	[62]
Human-*Bacteroides*	qBac560F qBac725R	qPCR	55	[63]
Zootechnical-*Bacteroides*	HF185F qHF183rnew	qPCR	58	[55]
amoA marker	amoA-1F amoA-1R	both PCR and qPCR	57	[60]
